# Prognostic and Clinicopathological Significance of E-Cadherin in Pancreatic Cancer Patients: A Meta-Analysis

**DOI:** 10.3389/fonc.2021.627116

**Published:** 2021-04-12

**Authors:** Pengbo Wang, Zengkuan Zhu

**Affiliations:** ^1^ Radiotherapy Department I, Yantaishan Hospital, Yantai, China; ^2^ Department of Oncology, Shengli Oilfield Central Hospital, Dongying, China

**Keywords:** E-cadherin, pancreatic cancer, susceptibility, prognosis, meta-analysis

## Abstract

**Background:**

Several recent studies have investigated the prognostic and clinicopathological significance of epithelial cadherin (E-cadherin) in pancreatic cancer; however, conclusions from these studies remain inconsistent. Therefore, we performed a meta-analysis to evaluate the effects of E-cadherin expression on the prognosis and clinicopathological characteristics of pancreatic cancer.

**Methods:**

Embase, PubMed, and Web of Science were searched to identify articles associated with E-cadherin and pancreatic cancer. Hazard ratios (HRs) and odds ratios (ORs) with corresponding confidence intervals (CIs) were calculated and summarized. All eligible studies were searched until May 20, 2020. Heterogeneity among studies was assessed using the Chi-square test and I^2^ statistic.

**Results:**

Overall, 25 studies were identified, of which 12 reports with 1,032 cases concerned the prognosis of pancreatic cancer, and 22 involved the risk and clinical characteristics of pancreatic cancer. The overall results revealed that E-cadherin expression was significantly related to overall survival, gender, tumor grade, lymph node metastasis, tumor differentiation, and risk of pancreatic cancer. In the subgroup analysis, no significant heterogeneity or publication bias was observed.

**Conclusions:**

E-cadherin expression is strongly associated with the risk, clinical features, and prognosis of pancreatic cancer, suggesting that E-cadherin may be an effective biomarker for the clinical assessments and predicting prognosis of pancreatic cancer.

## Introduction

Pancreatic cancer is one of the deadliest human malignancies, with a reported 5-year relative survival rate of less than 5% (1). According to published studies, approximately 88% of individuals diagnosed with pancreatic cancer are elderly patients (2). Meanwhile, 90% of newly diagnosed patients with pancreatic cancer are at an advanced stage, with 60% presenting lymph node metastasis, 69% involving nerve invasion, and free viable tumor cells detected in the peritoneal fluid of approximately 33% of patients (3, 4). Early detection could significantly improve the survival rate of pancreatic carcinoma, but early detection is hampered by several challenges. Prior to diagnosis, some commonly encountered symptoms associated with pancreatic cancer include loss of weight, nausea, indigestion or pain, abdominal or back pain, fatigue, or diabetes. However, these symptoms are often associated with several other conditions; thus, patients hardly ever visit a hospital or consider that they could be presenting signs of cancer. Frequently, only extreme pain or symptoms such as jaundice motivate patients to visit a physician, with tumor cells having metastasized at this stage. Although radiation therapy and chemotherapy are common therapeutic strategies during pancreatic cancer, surgery is the only potential cure for pancreatic cancer. However, patients who undergo surgery are known to ultimately present local or metastatic recurrence (5). Considering the poor prognosis and difficulties in early detection, novel detection and prognostic markers need to be identified. In recent years, investigations have focused on the effects of epithelial-mesenchymal transition (EMT) on pancreatic cancer. EMT is a process in which epithelial cells acquire motile mesenchymal features and promote cell migration and invasion (6). Accumulating evidence has revealed that EMT is closely related to the invasion and metastasis of pancreatic cancer (6, 7).

Epithelial cadherin (E-cadherin) is a typical type I classical cadherin, which is expressed mainly in epithelial cells and can mediate cell-cell interactions (8). Published studies have revealed that loss of E-cadherin can promote tumor cell invasiveness and correlates with poor prognosis of cancers (9, 10). Somatic mutations, silencing of the E-cadherin gene promoter, chromosomal deletions, or proteolytic cleavage of E-cadherin could lead to the suppression of E-cadherin expression, which is often detected in human tumors (11, 12). A recent study has reported that E-cadherin expression was downregulated in pancreatic tumor cells and correlated with tumor metastasis (13). Reportedly, the level of cadherin expression affects adhesion among cells. In contrast, E-cadherin inactivation may be involved in the differentiation of pancreatic tumor cells. However, several factors, including sample size, gender, and cancer stage, limit the credibility of observed results. To accurately elucidate the role of E-cadherin in the clinical progress and prognosis of pancreatic cancer, we collected published data to perform a meta-analysis.

## Methods

### Publication Search

Eligible reports regarding the association of E-cadherin expression with clinicopathological features and prognosis of pancreatic cancer were systematically searched in electronic databases, including PubMed, Embase, and Web of Science, until May 20, 2020. We searched these electronic databases using the following keywords: “pancreatic cancer,” “pancreatic carcinoma,” “E-cadherin,” “cadherin,” “CDH1” and “prognosis.” Two independent investigators collected the included studies based on the inclusion and exclusion criteria. Furthermore, the references cited in included studies were screened to identify other eligible studies.

### Inclusion and Exclusion Criteria

Eligible studies were included according to predefined inclusion and exclusion criteria. All included studies had to meet the following inclusion criteria: (1) studies published in English; (2) case-control studies, retrospective studies, or cohort studies; (3) patients had to present detailed pathological diagnostic criteria; (4) studies had to detail sufficient data to calculate hazard ratios (HRs), odds ratios (ORs), and 95% CIs. Conversely, studies were excluded based on the following exclusion criteria: (1) reviews, comments, letters, conferences, case reports, editorials, and meta-analyses; (2) studies with duplicated data; (3) no available data.

### Data Extraction and Quality Assessment

Data regarding the relationship between E-cadherin expression and risk, prognosis, and clinicopathological parameters of pancreatic cancer were extracted by two researchers. The following information was collected from each study: first author’s name, publication year, country, ethnicity, protein expression detection method, cancer subtype, sample type, HRs and 95% confidence intervals (CIs), survival curve, and the number of cases and controls in different groups. The Newcastle–Ottawa Scale (NOS) table (http://www.ohri.ca/programs/clinical_epidemiology/oxford.asp, accessed on June 1, 2019) was applied to comprehensively evaluate the quality of included studies that involved the relationship between the risk of pancreatic cancer and E-cadherin expression. The included studies were categorized as low (<4), medium (4–6), or high (>6) according to their quality scores (14).

### Statistical Analysis

Statistical analyses were performed using STATA Statistical Software (Version 14.2; StataCorp LP, College Station, TX, USA). Initially, HRs, ORs, 95% CIs, and the weight of each included study were calculated with a fixed-effect model and random-effects model to estimate the role of E-cadherin expression in risk, prognosis, and clinicopathological features of pancreatic cancer (15). Statistical significance was set at P < 0.05. The Chi-square test and I^2^ statistic were used to evaluate the significance of heterogeneity among the studies (16). If significant heterogeneity was detected, the random-effects model (DerSimonian–Laird method) was applied; otherwise, the fixed-effect model (the Mantel-Haenszel method) was used (15). Begg’s rank test and Egger’s regression test were used for evaluating the publication bias of each study, which could be visually measured by the asymmetry of Begg’s funnel plot (17). In addition, the leave-one-out sensitivity analysis was performed to further assess the effects of individual studies on overall results (18). Finally, a meta-analysis was performed in accordance with the Preferred Reporting Items for Systematic Reviews and Meta-Analyses (PRISMA).

## Results

### Search Strategy and Selection

**Figure 1:**
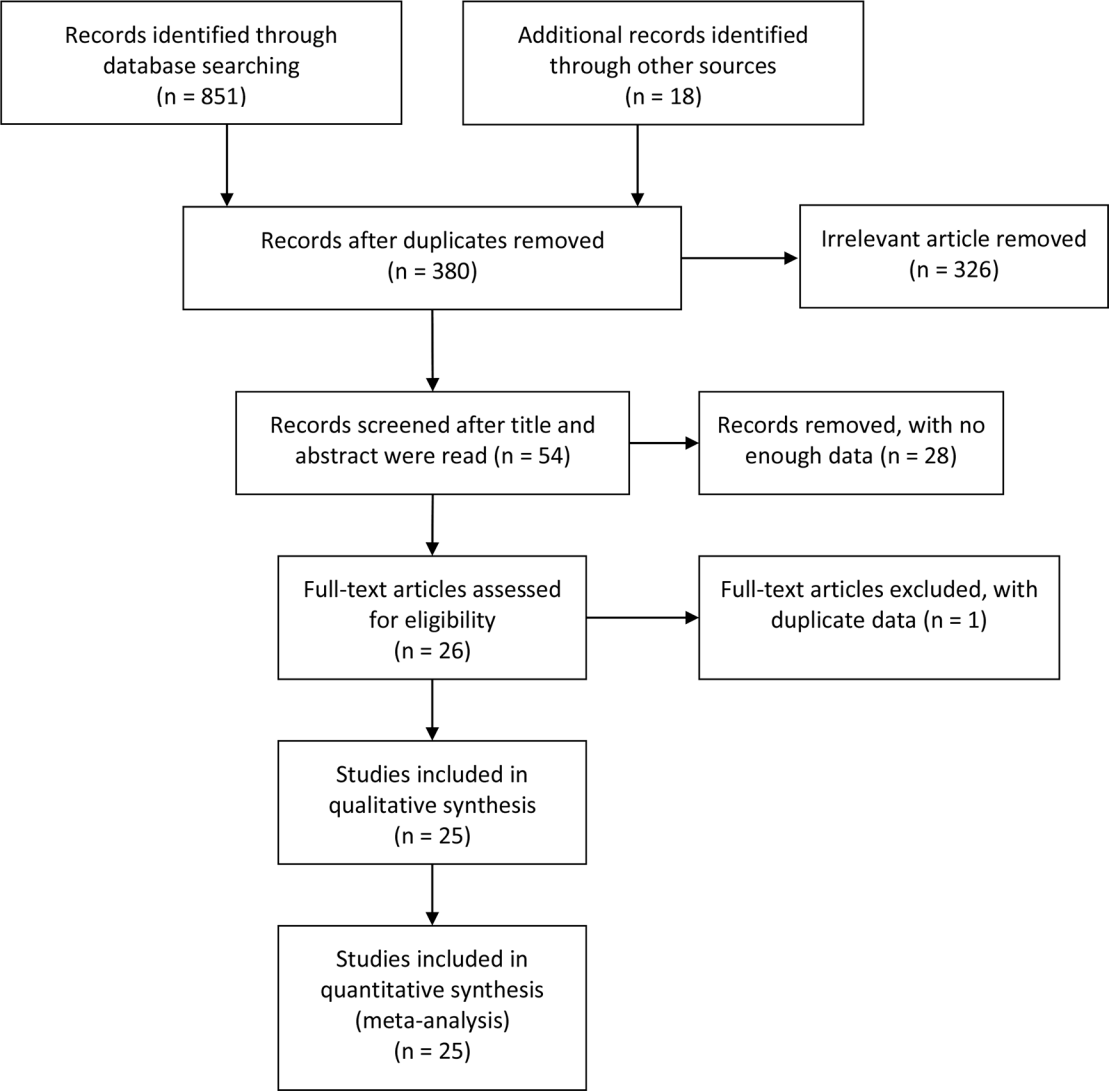
Flow chart for literature search.

Of the 381 studies initially identified, 12 studies involving 1,032 pancreatic cancer patients analyzed the influence of E-cadherin on the prognosis of pancreatic cancer, whereas 22 studies assessed the risk and clinicopathological features of pancreatic cancer (19–43). However, the study by Wang et al. significantly affected the stability of pooled HRs and was excluded according to the sensitivity analysis (30). Therefore, in the final 11 studies on prognosis, three studies were reported in Caucasian populations (22, 27, 28), with eight concentrating on Asian populations (19–21, 23–26, 29). Furthermore, four reports were collected to assess the risk of pancreatic cancer (24, 25, 28, 31), while 18 studies were included in the analysis of clinicopathological features of pancreatic cancer. In all eligible studies, E-cadherin expression was detected using immunohistochemistry (IHC). Seven studies directly provided HR estimates and 95% CIs, while other studies reported survival curves in which Engauge Digitizer 4.1 software was applied to extracted HRs and 95% CIs (**Tables 1** and **2**) (**Figure 1**).

**Table 1:** Characteristics of eligible studies in the meta-analysis for the OS of pancreatic cancer.

Author	References	Time	Country	Ethnicity	Tumor stage	Cancer	Number	Follow-up time	Survival analysis	Source of HR	HRs	LL	UL	95%CI	*P*	Cut-off value
Shimamura et al.	19	2003	Japan	Asians	IHC	PDCA	125	23.2 months	OS	HR	1.82	1.17	2.83	1.17-2.83	0.008	20%
Shin et al.	20	2005	Korea	Asians	IHC	PC	53	15.6 months	OS	Curve	2.63	0.82	3.79	0.82-3.79	0.3098	10%
Oida et al.	21	2006	Japan	Asians	IHC	PC	72	NR	OS	HR	1.264	0.733	2.18	0.733-2.180	0.399	NR
Hong et al.	22	2011	USA	Caucasians	IHC	PDCA	323	12.1 months	OS	HR	6.34	2.5	16.1	2.5-16.1	0.001	5%
Xu et al.	23	2013	China	Asians	IHC	PC	60	11.93 months	OS	Curve	3.53	2.02	4.85	2.02-4.85	0.006	70%
Jiao et al.	24	2016	China	Asians	IHC	PC	84	30.2 months	OS	HR	1.96	1.07	3.89	1.07-3.89	0.012	10%
Chen et al.	25	2017	China	Asians	IHC	PC	80	34.8 months	OS	HR	2.02	0.87	3.94	0.87-3.94	0.057	10%
Chang et al.	26	2017	China	Asians	IHC	IPMNPC	87	46 months	OS	HR	13.718	2.28	82.519	2.28-82.519	0.004	NR
Grupp et al.	27	2018	Germany	Caucasians	IHC	PDCA	34	45 months	OS	Curve	2.15	0.87	3.48	0.87-3.48	0.375	NR
Radulovic and Kruslin	28	2018	Croatia	Caucasians	IHC	PDCA	61	NR	OS	Curve	1.56	1.13	2.93	1.13-2.93	0.02	5%
Noda et al.	29	2019	Japan	Asians	IHC	PDCA	53	16 months	OS	Curve	2.25	1.69	3.26	1.69-3.26	0.017	NR
Wang et al.	30	2019	China	Asians	IHC	PC	1.2	14.4 months	OS	HR	22.9	18.9	26.8	18.9-26.8	0.005	75%

PDCA, pancreatic ductal adenocarcinoma; PC, pancreatic cancer; IPMNPC, intraductal papillary mucinous neoplasm of pancreatic cancer; OS, overall survival; NR, not reported; IHC, immunohistochemistry.

**Table 2:** Characteristics of eligible studies in the meta-analysis for the clinical features of pancreatic cancer.

Author	Reference	Time	Country	Ethnicity	Method	Histology	Absent		Present		Cut-off
							E-cadherin+	E-cadherin-	E-cadherin+	E-cadherin-	
Lymph node metastasis											
Karayiannakis et al.	37	1998	Greece	Caucasians	IHC	PC	15	4	10	14	10%
Joo et al.	32	2002	Korea	Asians	IHC	PDCA	11	8	1	10	10%
Shimamura et al.	19	2003	China	Asians	IHC	PDCA	8	7	59	51	20%
Nakajima et al.	39	2004	Japan	Asians	IHC	PC	4	5	9	12	10%
Shin et al.	20	2005	Korea	Asians	IHC	PC	17	23	7	6	10%
Oida et al.	21	2006	Japan	Asians	IHC	PDCA	21	26	4	9	NR
Torer et al.	40	2007	Turkey	Caucasians	IHC	PA	14	6	7	1	NR
Pryczynicz et al.	31	2010	Poland	Caucasians	IHC	PDCA	7	12	2	8	50%
Hong et al.	22	2011	USA	Caucasians	IHC	PDCA	30	16	158	125	5%
Kurahara et al.	33	2012	Japan	Asians	IHC	PC	19	10	12	35	10%
Gu et al.	34	2013	China	Asians	IHC	PDCA	14	14	2	12	5%
Guo et al.	35	2014	China	Asians	IHC	PA	27	21	9	19	10%
Jiao et al.	24	2016	China	Asians	IHC	PC	37	14	16	17	10%
Chen et al.	25	2017	China	Asians	IHC	PC	30	17	18	15	10%
Radulovic and Kruslin	28	2018	Croatia	Caucasians	IHC	PDCA	7	18	8	22	5%
Wang et al.	30	2019	USA	Caucasians	IHC	PDCA	32	8	63	17	10%
Noda et al.	29	2019	Japan	Asians	IHC	PDCA	24	27	0	2	NR
Differentiation							Poor		Moderate and Well		
							E-cadherin+	E-cadherin-	E-cadherin+	E-cadherin-	
Yonemasu et al.	43	2001	Japan	Asians	IHC	PDCA	1	7	4	1	10%
Joo et al.	32	2002	Korea	Asians	IHC	PDCA	1	7	11	11	10%
Watanabe et al.	42	2003	Japan	Asians	IHC	PDCA	0	7	5	11	10%
Hong et al.	22	2011	USA	Caucasians	IHC	PDCA	72	74	116	67	5%
Gu et al.	34	2013	China	Asians	IHC	PDCA	3	14	13	12	5%
Jiao et al.	24	2016	China	Asians	IHC	PC	17	10	36	21	10%
Chen et al.	25	2017	China	Asians	IHC	PC	18	8	30	24	10%
Wang et al.	30	2019	USA	Caucasians	IHC	PDCA	30	14	65	11	10%

PDCA, pancreatic ductal adenocarcinoma; PC, pancreatic cancer; PA, pancreatic adenocarcinoma; NR, not reported; IHC, immunohistochemistry.

### Correlation Between E-Cadherin Expression and Prognosis of Pancreatic Cancer

**Figure 2:**
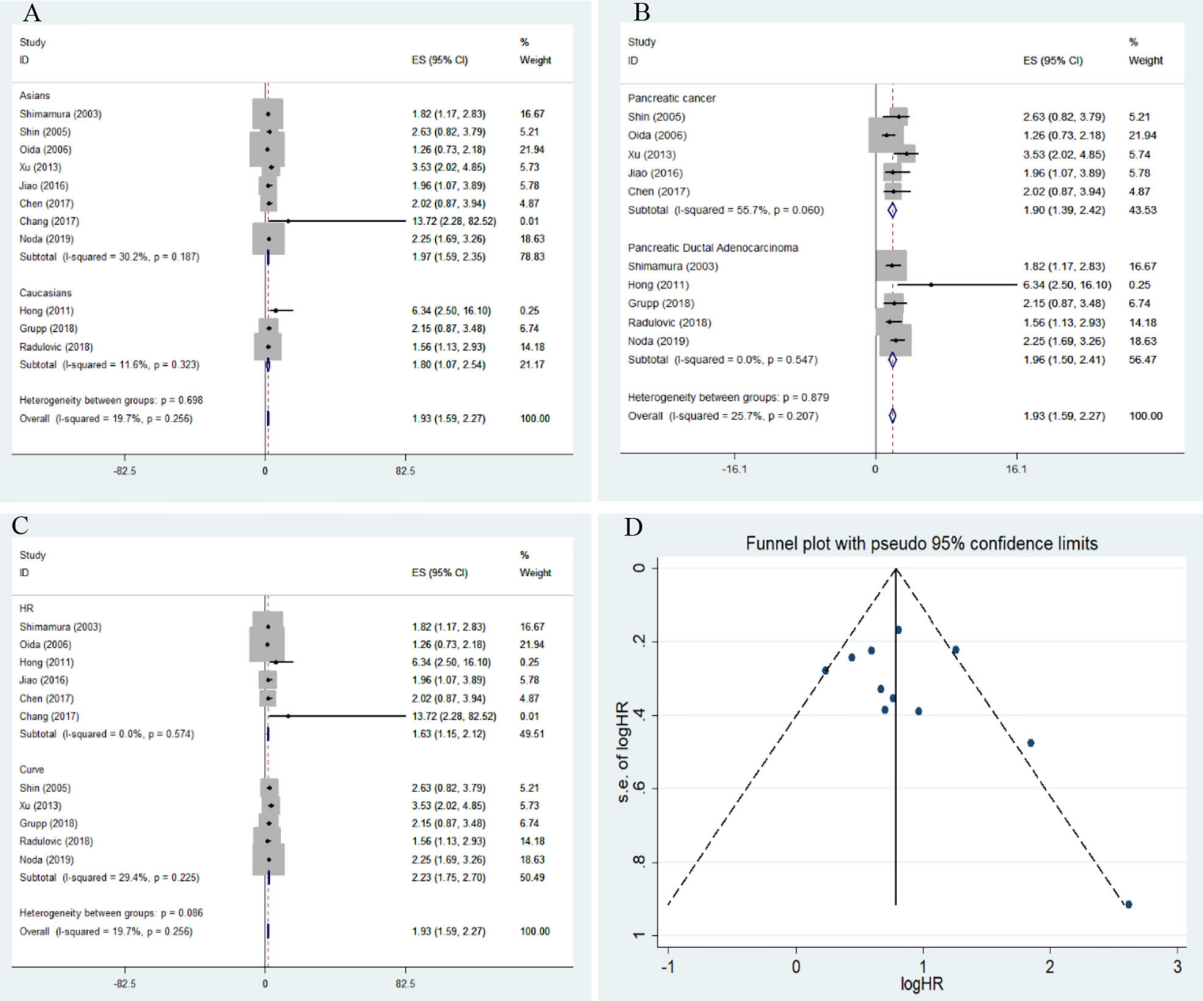
Forest plot and funnel plot for the association between E-cadherin expression and overall survival of pancreatic cancer. **(A)** subgroup analysis for ethnicity; **(B)** subgroup analysis for cancer subtype; **(C)** subgroup analysis for the source of hazard ratios (HRs); **(D)** funnel plot for the association between E-cadherin expression and overall survival of pancreatic cancer.

The overall results indicated a significant relationship between E-cadherin expression and overall survival (OS) of pancreatic cancer (HR = 1.93, 95% CI = 1.59-2.27, P < 0.05). No significant heterogeneity was detected; therefore, a fixed-effects model was used to calculate the pooled HRs. The pooled HR was 1.93, indicating that decreased expression levels of E-cadherin were significantly associated with the poor OS of pancreatic cancer. To further assess the association between aberrant E-cadherin expression and OS in pancreatic cancer, subgroup analyses based on race, source of HRs, and cancer subtype were conducted. The subgroup analysis showed similar results both in Caucasian (HR = 1.80, 95% CI = 1.07-2.54, P < 0.05) and Asian populations (HR = 1.97, 95% CI = 1.59-2.35, P < 0.05). In addition, the subgroup analysis for pancreatic ductal adenocarcinoma (PDCA) revealed that low E-cadherin expression led to a poor OS in pancreatic cancer (HR = 1.96, 95% CI = 1.50-2.41, P < 0.05). None of the subgroup analyses detected significant heterogeneity across studies (**Table 3**) (**Figure 2**).

**Table 3:** Meta-analysis result of pooled HR for overall survival in pancreatic cancer patients.

Characteristics (Positive vs Negative)	Studies	Number	Pooled HR (95% CI)	*P*	Heterogeneity		Begg's test		Egger's test	
					I^2^ (%)	*P*	Z	*P*	T	*P*
PC										
OS	11	1032	1.93 (1.59-2.27)	<0.05	20%	0.256	1.17	0.243	1.28	0.234
OS in Caucasians	3	418	1.80 (1.07-2.54)	<0.05	12%	0.323	1.57	0.117	3.17	0.195
OS in Asians	8	614	1.97 (1.59-2.35)	<0.05	30.20%	0.187	0.74	0.458	0.67	0.526
OS (HR)	6	771	1.63 (1.15-2.12)	<0.05	0.00%	0.574	1.88	0.06	2.85	0.05
OS (Survival curve)	5	261	2.23 (1.75-2.70)	<0.05	29.40%	0.225	0.49	0.625	-0.02	0.987
PDCA										
OS	5	596	1.96 (1.50-2.41)	<0.05	0.00%	0.547	0.49	0.624	1.03	0.379

PDCA, pancreatic ductal adenocarcinoma; PC, pancreatic cancer; OS, overrall survival; HR, hazard ratio.

### Correlation Between E-Cadherin Expression and Clinicopathological Features of Pancreatic Cancer

Among the included studies, four investigated the association between E-cadherin expression and the risk of pancreatic cancer, and a significant association was observed among Caucasian and Asian populations (overall results, OR = 6.70, 95% CI = 2.25-19.95, P < 0.05; Caucasian, ORs = 38.84, 95% CI = 2.71-556.57, P < 0.05; Asian, ORs = 4.01, 95% CI = 2.32-6.95, P < 0.05). Therefore, decreased E-cadherin expression may lead to an increased risk of pancreatic cancer. Next, we assessed the correlation between decreased E-cadherin expression and the clinicopathological features of patients with pancreatic cancer. The results are summarized in **Table 4**. As expected, decreased E-cadherin expression was significantly correlated with poor differentiation of pancreatic cancer (overall results, ORs = 0.55, 95% CI = 0.40-0.76, P < 0.05), which was observed in Caucasian (OR = 0.52, 95% CI = 0.35-0.77, P < 0.05) but not in Asian populations (Asian, OR = 0.62, 95% CI = 0.37-1.05, P > 0.05). In addition, we noted that decreased E-cadherin expression was significantly associated with tumor grade (overall results, ORs = 2.50, 95% CI = 1.40-3.00, P < 0.05; Caucasian, OR = 1.38, 95% CI = 0.69-2.75, P > 0.05; Asian, ORs = 2.44, 95% CI = 1.54-3.86, P < 0.05) and lymph node metastasis (overall results, OR = 1.88, 95% CI = 1.43-2.46, P < 0.05; Caucasian, OR = 1.47, 95% CI = 0.96-2.25, P > 0.05; Asian, ORs = 2.22, 95% CI = 1.56-3.15, P < 0.05), which indicated that decreased E-cadherin expression predicted poor clinical outcomes of pancreatic cancer. However, no statistical correlations were observed between E-cadherin expression and TNM stage (OR = 1.28, 95% CI = 0.91-1.81, P > 0.05) or vascular invasion of pancreatic cancer (OR = 1.01, 95% CI = 0.71-1.44, P > 0.05), both in Caucasian and Asian populations. Although the overall results revealed that E-cadherin expression was significantly associated with metastasis of pancreatic cancer, no significant association was observed in the subgroup analysis based on ethnicity (overall results, OR = 1.58, 95% CI = 1.04-2.39, P < 0.05; Caucasian, OR = 3.02, 95% CI = 0.68-13.31, P > 0.05; Asian, OR = 1.48, 95% CI = 0.96-2.29, P > 0.05). . Heterogeneity was detected in the analysis for the differentiation of pancreatic cancer, and subgroup analysis found no significant heterogeneity in the subgroup analysis of Caucasian populations. Heterogeneity in Asian populations revealed that other factors might affect the accuracy of results for differentiation. Moreover, the incidence of decreased E-cadherin expression in males (51.94%) was higher than that in females (43.24%) (overall, OR = 1.61, 95% CI = 1.19-2.17, P < 0.05), which was also observed in the stratified analysis based on ethnicity.

**Table 4:** Meta-analysis result of pooled ORs for clinical features of pancreatic cancer patients.

Characteristics (Positive vs Negative)	Study	Pooled ORs (95% CI)	*P*	Heterogeneity		Begg's test		Egger's test	
				I^2^ (%)	*P*	Z	*P*	T	*P*
Risk (Overall)	4	6.70 (2.25-19.95)	<0.05	62.10	0.048	1.36	0.174	2.15	0.165
Risk (Caucasians)	2	38.84 (2.71-556.57)	<0.05	42.00	0.189	-1.00	0.317	–	–
Risk (Asians)	2	4.01 (2.32-6.95)	<0.05	0.00	0.611	1.00	0.317	–	–
Gender (Female vs Male)	12	1.61(1.19-2.17)	<0.05	18.40	0.263	0.82	0.411	-0.20	0.846
Gender (Caucasians) (Female vs Male)	3	2.14 (1.06-4.30)	<0.05	48.60	0.143	0.52	0.602	0.09	0.940
Gender (Asians) (Female vs Male)	9	1.50 (1.08-2.10)	<0.05	12.20	0.333	0.63	0.532	-0.65	0.535
Tumor grade (Overall) (G1 vs G2+G3)	11	2.50 (1.40-3.00)	<0.05	37.90	0.096	0.08	0.938	0.35	0.736
Tumor grade (Caucasians) (G1 vs G2+G3)	5	1.38 (0.69-2.75)	>0.05	47.00	0.109	0.00	1.000	0.15	0.890
Tumor grade (Asians) (G1 vs G2+G3)	6	2.44 (1.54-3.86)	<0.05	25.40	0.244	0.56	0.573	1.07	0.344
Lymph node metastasis (Overall) (N0 vs N1)	17	1.88 (1.43-2.46)	<0.05	30.30	0.115	0.58	0.564	0.68	0.508
Lymph node metastasis (Caucasians) (N0 vs N1)	6	1.47 (0.96-2.25)	>0.05	14.60	0.321	-0.19	0.851	-0.05	0.962
Lymph node metastasis (Asians) (N0 vs N1)	11	2.22 (1.56-3.15)	<0.05	34.80	0.120	0.54	0.586	0.66	0.524
TNM stage (Overall) (T1+T2 vs T3+T4)	12	1.28 (0.91-1.81)	>0.05	31.00	0.143	0.00	1.000	-0.26	0.803
TNM stage (Caucasians) (T1+T2 vs T3+T4)	3	2.04 (0.95-4.36)	>0.05	30.30	0.238	0.52	0.602	0.06	0.960
TNM stage (Asians) (T1+T2 vs T3+T4)	9	1.13 (0.77-1.67)	>0.05	31.00	0.170	-0.42	0.677	-0.51	0.626
Metastasis (Overall) (M0 vs M1)	12	1.58 (1.04-2.39)	<0.05	0.00	0.627	1.95	0.052	0.66	0.523
Metastasis (Caucasians) (M0 vs M1)	2	3.02 (0.68-13.31)	>0.05	23.40	0.253	1.00	0.317	–	–
Metastasis (Asians) (M0 vs M1)	10	1.48 (0.96-2.29)	>0.05	0.00	0.619	1.04	0.297	0.07	0.945
Vascular invasion (Overall) (Absent vs Present)	5	1.01 (0.71-1.44)	>0.05	0.00	0.421	0.49	0.624	0.08	0.939
Vascular invasion (Caucasians) (Absent vs Present)	2	0.98 (0.64-1.52)	>0.05	0.00	0.343	0.52	0.602	0.93	0.523
Vascular invasion (Asians) (Absent vs Present)	3	1.06 (0.58-1.92)	>0.05	32.80	0.226	-1.00	0.317	–	–
Differentiation (Overall) (Poor vs Well)	8	0.55 (0.4-0.76)	<0.05	53.1	0.04	-0.99	0.322	-1.32	0.236
Differentiation (Caucasians) (Poor vs Well)	2	0.52 (0.35-0.77)	<0.05	0	0.39	-1.00	0.317	–	–
Differentiation (Asians) (Poor vs Well)	6	0.62 (0.37-1.05)	>0.05	63.4	0.02	-0.94	0.348	-3.54	0.024

TNM, T: Tumor, N: Lymph Node, M: Metastasis.

**Figure 3:**
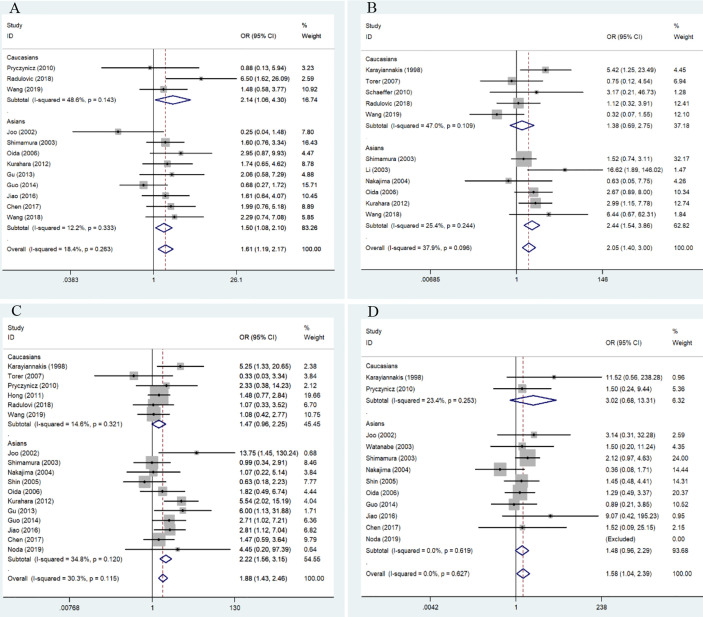
Forest plot for the association of E-cadherin expression with gender, grade, lymph node metastasis, and differentiation of patients with pancreatic cancer. **(A)** gender; **(B)** tumor grade; **(C)** lymph node metastasis; **(D)** differentiation.

We further performed a subgroup analysis to evaluate clinical feature data for PDCA, and these results revealed that downregulation of E-cadherin was associated with gender (overall results, OR = 1.73, 95% CI = 1.14-2.63, P < 0.05; Caucasian, ORs = 2.14, 95% CI = 1.06-4.30, P < 0.05), lymph node metastasis (Overall results, ORs = 1.66, 95% CI = 1.14-2.43, P < 0.05; Asian, ORs = 2.44, 95% CI = 1.28-4.67, P < 0.05) and metastasis (overall results, OR = 1.76, 95% CI = 1.03-3.01, P < 0.05), and differentiation (Overall results, ORs = 0.44, 95% CI = 0.30-0.63, P < 0.05; Caucasian, ORs = 0.52, 95% CI = 0.35-0.77, P < 0.05; Asian, ORs = 0.14, 95% CI = 0.05-0.45, P < 0.05) in pancreatic cancer. Based on Cochran’s Q and I^2^ statistics, heterogeneity was not detected among included studies (**Table 5**) (**Figure 3**).

**Table 5:** Meta-analysis results of pooled ORs for clinical features in PDCA patients.

Characteristics (Positive vs Negative)	Studies	Pooled ORs (95% CI)	*P*	Heterogeneity		Begg's test		Egger's test	
				I^2^ (%)	*P*	Z	*P*	T	*P*
Gender (Female vs Male)	7	1.73 (1.14-2.63)	<0.05	36.60	0.149	-0.45	0.652	-0.40	0.707
Gender (Caucasians) (Female vs Male)	3	2.14 (1.06-4.30)	<0.05	48.60	0.143	0.52	0.602	0.09	0.940
Gender (Asians) (Female vs Male)	4	1.53 (0.91-2.57)	>0.05	43.60	0.150	-0.68	0.497	-0.70	0.558
Tumor grade (Overall) (G1 vs G2+G3)	5	1.44 (0.87-2.38)	>0.05	23.50	0.265	-0.49	0.624	-0.32	0.771
Tumor grade (Caucasians) (G1 vs G2+G3)	3	0.85 (0.34-2.13)	>0.05	22.30	0.276	0.52	0.602	0.40	0.760
Tumor grade (Asians) (G1 vs G2+G3)	2	1.80 (0.87-2.38)	>0.05	0.00	0.398	1.00	0.317	–	–
Lymph node metastasis (Overall) (N0 vs N1)	9	1.66 (1.14-2.43)	<0.05	6.40	0.382	2.29	0.022	2.35	0.050
Lymph node metastasis (Caucasians) (N0 vs N1)	4	1.35 (0.84-2.16)	>0.05	0.00	0.850	0.68	0.497	0.17	0.878
Lymph node metastasis (Asians) (N0 vs N1)	5	2.44 (1.28-4.67)	<0.05	37.60	0.170	1.47	0.142	2.27	0.108
TNM stage (Overall) (T1+T2 vs T3+T4)	5	0.75 (0.39-1.46)	>0.05	21.70	0.276	0.49	0.624	0.68	0.543
Metastasis (Overall) (M0 vs M1)	6	1.76 (1.03-3.01)	<0.05	0.00	0.923	0.49	0.624	0.04	0.971
Differentiation (Overall) (Poor vs Well)	5	0.44 (0.30-0.63)	<0.05	34.20	0.193	-1.96	0.050	-11.35	0.001
Differentiation (Caucasians) (Poor vs Well)	2	0.52 (0.35-0.77)	<0.05	0.00	0.392	-1.00	0.317	–	–
Differentiation (Asians) (Poor vs Well)	3	0.14 (0.05-0.45)	<0.05	0.00	0.609	-1.57	0.117	-2.49	0.243

PDCA, pancreatic ductal adenocarcinoma; TNM, T: Tumor, N: Lymph Node, M: Metastasis.

### Publication Bias and Sensitivity Analysis

Sensitivity analysis was performed to examine the effects of each included study on pooled HRs and ORs. For OS in pancreatic cancer, the results of Wang et al. (30) significantly affected the pooled HRs; accordingly, we removed this study from all meta-analyses for the OS of pancreatic cancer. In the other subgroup analysis for risk and clinical features, no individual study significantly altered the pooled ORs; however, heterogeneity was still observed in the analysis for differentiation in Asian populations, which indicated that other factors might have led to the main source of heterogeneity. Egger’s test and Begg’s funnel plot revealed the absence of publication bias in the analysis.

## Discussion

During EMT, the cobblestone epithelial appearance of cultured epithelial cells gradually changed to a spindle-shaped, mesenchymal morphology. This transformation is reversible, in which mesenchymal cells can convert back into an epithelial state, known as a mesenchymal–epithelial transition (MET) (44). EMT plays a crucial role in embryogenesis and tissue morphogenesis; therefore, several studies focused on the relationship between EMT, as well as development and wound healing in adults (45). Normally, epithelial cells in tissues are connected through tight junctions and adherens junctions, and the adherens junctions are linked to cell surface E-cadherin molecules. Increasing evidence indicates that epigenetic changes regulate EMT, which is not dependent on DNA sequence alterations in normal and cancer cells (46). Furthermore, the identified somatic mutations and hypermethylation of the E-cadherin promoter did not result in its downregulation (47). Reportedly, recruitment of the histone deacetylases, HDAC1 and HDAC2, by the transcriptional repressor ZEB1 might be responsible for changes in E-cadherin expression (47). Following the activation of EMT, the expression of E-cadherin is repressed, which weakens the connection between cells and leads to the loss of typical polygonal, cobblestone morphology of epithelial cells (48). The activation of the EMT program often works in association with other signaling pathways to promote the conversion of epithelial cells into a fully mesenchymal state in normal cells, which is also observed in cancer cells (49). As one of the key regulators of EMT, E-cadherin may play an important role in primary tumor development and progression. Decreased expression of E-cadherin might reduce the adhesive strength and suppress the function of downstream transcription factors such as snail, LEF-1, and ZEB1.

In the present meta-analysis, the pooled results suggested that cancer tissues present a lower E-cadherin expression than normal tissues or adjacent tissues (24, 25, 28, 31), which indicated that EMT occurred during pancreatic cancer carcinogenesis. E-cadherin expression was significantly correlated with worse histopathological grade (19, 21, 28, 30, 33, 36–41), lymphatic invasion-positive tumor (19–22, 24, 25, 28–35, 37, 39, 40), and poor differentiation of pancreatic cancer (22, 24, 25, 30, 32, 34, 42, 43), which revealed that E-cadherin expression was significantly linked to malignant biological behaviors and tumor progression. Moreover, the pooled HR was 1.80 in Caucasian and 1.97 in Asian populations for OS, revealing that decreased E-cadherin expression predicted poorer prognosis and might be a potential biomarker for estimating the survival rate in pancreatic cancer patients. Furthermore, the number of male patients with pancreatic cancer presenting decreased E-cadherin expression was higher than female patients, and different lifestyle factors such as smoking and drinking in men could be responsible for these findings.

Tissue samples from different subtypes of pancreatic tumors were included in the current meta-analysis. Therefore, subgroup analysis based on cancer type was performed to investigate the association between E-cadherin expression and PDCA, which is the most frequent type of pancreatic cancer. A significant correlation was detected between E-cadherin downregulation and the prognosis and clinical progress of PDCA. Furthermore, we observed a more obvious association between E-cadherin downregulation and poor PDCA differentiation. In addition, according to the pooled ORs, E-cadherin downregulation promoted the metastasis of PDCA cells. These observations were in line with the perspective that E-cadherin is a tumor suppressor molecule in cancer development (50), which further highlighted that the EMT process is pivotal in cancer invasion and metastasis (3). Accordingly, E-cadherin may be a useful therapeutic target for PDCA to inhibit cancer progression. Wang et al. have observed that the level of E-cadherin expression gradually decreases over time with microenvironmental changes, indicating that the microenvironment might reduce E-cadherin expression (36).

There is growing evidence that the tumor microenvironment (TME) plays an important role in the progression and prognosis of pancreatic cancer. The TME consists of the extracellular matrix (ECM), aberrant immune cells, pancreatic stellate cells, and abnormal tumor vascularization (51). It has been reported that E-cadherin mutations and aberrant downregulation of E-cadherin can be observed in breast cancer, which might be associated with an abnormal TME (52). In the present study, E-cadherin expression was significantly associated with grade, metastasis, and differentiation of pancreatic cancer. In a published study, E-cadherin was significantly associated with lymph node metastasis in gastric cancer (53). Cancer-associated fibroblasts could lead to the deposition of the ECM and mediate the process of EMT, which includes regulation of E-cadherin expression level (54). Therefore, it might be crucial to clarify the level of E-cadherin expression in different cells in future studies.

Based on findings of the current meta-analysis, E-cadherin expression was significantly decreased in pancreatic cancer tissue samples and presented a close relationship with poor prognosis and malignant progression of the tumor patients. In the analysis of pancreatic cancer differentiation, we detected heterogeneity among studies of Asian populations; accordingly, subgroup analysis based on cancer type was further performed in PDCA. No heterogeneity was observed in the analysis of PDCA. Additionally, the sensitivity analysis results revealed that no individual study significantly affected the stability of the pooled HRs and ORs.

Although heterogeneity and publication bias were rarely observed, some limitations persist in the present study. First, we extracted HRs from the survival curve when eligible studies did not directly provide HRs, and the extracted HRs might minimally differ from the real data. Second, the sample number in some subgroup analyses was small. Third, although we included all eligible literature in English, several studies in other languages were not included, which might have excluded some potential data.

In conclusion, this meta-analysis demonstrated that decreased E-cadherin expression was positively correlated with advanced clinical features and poor OS in patients with pancreatic cancer, suggesting that it might be a potential biomarker for clinical assessments and predicting the prognosis of pancreatic cancer.

## Data Availability Statement

The original contributions presented in the study are included in the article/**Supplementary Material**. Further inquiries can be directed to the corresponding author.

## Author Contributions

All authors listed have made a substantial, direct, and intellectual contribution to the work and approved it for publication.

## Conflict of Interest

The authors declare that the research was conducted in the absence of any commercial or financial relationships that could be construed as a potential conflict of interest.

## Abbreviations

HRs, Hazard ratios; ORs, Odds ratios; CIs, confidence intervals; PDCA, pancreatic ductal adenocarcinoma; PC, pancreatic cancer; IPMNPC, intraductal papillary mucinous neoplasm of pancreatic cancer; OS, overall survival; NR, not reported; PA, pancreatic adenocarcinoma; IHC, immunohistochemistry.
