# Supplementary material for: Prognostic and Clinicopathological Significance of E-Cadherin in Pancreatic Cancer Patients: A Meta-Analysis
Source: Front Oncol. 2021 Apr 12;11:627116. doi: 10.3389/fonc.2021.627116 (PMC8074677; doi:10.3389/fonc.2021.627116)
Supplement: Supplementary file 2 [file Table_2.doc]

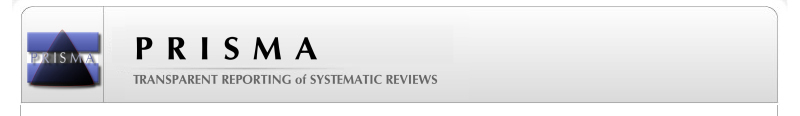
**PRISMA 2009 Flow Diagram**

**Screening**

**Included**

**Eligibility**

**Identification**

Records identified through database searching
(n = 851)

Additional records identified through other sources
(n = 18)

Records after duplicates removed
(n = 381)

Records screened after title and abstract were read (n = 54)

Records removed, with no enough data (n = 28)

Full-text articles assessed for eligibility
(n = 26)

Full-text articles excluded, with duplicate data (n = 1)

Studies included in qualitative synthesis
(n = 25)

Studies included in quantitative synthesis (meta-analysis)
(n = 25)

Irrelevant article removed
(n = 327)
